# Some explicit solutions of the three-dimensional Euler equations with a free surface

**DOI:** 10.1007/s00208-021-02323-2

**Published:** 2021-12-17

**Authors:** Calin I. Martin

**Affiliations:** Faculty of Mathematics, University of Vienna, Oskar-Morgenstern-Platz 1, 1090 Vienna, Austria

**Keywords:** 35Q31, 35Q35, 76B15, 76B70

## Abstract

We present a family of radial solutions (given in Eulerian coordinates) to the three-dimensional Euler equations in a fluid domain with a free surface and having finite depth. The solutions that we find exhibit vertical structure and a non-constant vorticity vector. Moreover, the flows described by these solutions display a density that depends on the depth. While the velocity field and the pressure function corresponding to these solutions are given explicitly through (relatively) simple formulas, the free surface defining function is specified (in general) implicitly by a functional equation which is analysed by functional analytic methods. The elaborate nature of the latter functional equation becomes simpler when the density function has a particular form leading to an explicit formula of the free surface. We subject these solutions to a stability analysis by means of a Wentzel–Kramers–Brillouin (WKB) ansatz.

## Introduction

The momentum conservation equations together with the equation of mass conservation (proposed by Euler in the middle of the eighteenth century) are widely used today when tackling fluid flow problems. In spite of tremendous progress made over the past two and a half centuries many important questions concerning fluids still remain unanswered. Along the previous lines we note, for instance, the scarcity of explicit solutions. Motivated by this circumstance we set out for a study of the three-dimensional Euler equations with a free surface from the perspective of explicit solutions. While less significant for engineering applications, explicit solutions are interesting for a variety of reasons. Indeed, such solutions may play an appreciable role in the validation of the governing equations. On the other hand, in an oceanographical context, they provide the basis for physically more realistic flows by way of asymptotic, or perturbative methods [40]; the latter is also a line of work that we undertake here.

It is to note that the only known explicit solution to the nonlinear (two-dimensional) gravity water wave problem for an inviscid, incompressible and homogeneous flow of infinite depth was pointed out by Gerstner in 1809, cf. [25], rediscovered by Rankine [51] and describes the evolution of individual fluid particles in a flow: particles move on circles with radius decreasing with depth. The physical acceptability of the Gerstner’s solution was demonstrated by Constantin [4] who, availing of an interplay of analytical and topological ideas, showed that the motion suggested by Gerstner’s ansatz is dynamically possible. For in-depth studies of the Gerstner wave solution we refer the reader to [4, 27]. A family of explicit solutions for the case of water waves driven by surface tension was provided by Crapper [19]. These solutions were proved to be unique by Okamoto [48].

The Gerstner wave solution was extended by Constantin [10, 12] and adapted to bear relevance to the important case of geophysical water flows, that is, flows looked at from the perspective of a frame that rotates with the Earth. Further descriptions of geophysical flows based on extensions of the Gerstner’s wave were performed in [15, 29, 33, 45, 46]. The flow pattern of these solutions is given in Lagrangian coordinates and it is inherently three-dimensional, a typical feature displayed by geophysical flows, cf. [17].

The unsatisfactory situation regarding the lack of explicit solutions is mitigated by the availability of rigorous studies on the existence of exact solutions to the nonlinear water wave problem [2, 5, 14, 18, 26, 37, 38, 52, 54] as well as on qualitative properties of the solutions [6–8, 22, 49].

Certainly, the Lagrangian perspective offers important insights into the flow evolution by following the path of a particular particle, but, on the other hand, it is highly significant to know the velocity field, the pressure function and the shape of the free surface at any given time instant and physical location, setting known as the Eulerian picture. Complying with the previous ideas we present in this paper some explicit solutions (in Eulerian coordinates) to the nonlinear inviscid and incompressible three-dimensional water wave problem. While the Gerstner wave solution supplies explicit formulas for the velocity field and for the pressure function, (some) of the solutions presented here are explicit also in terms of the free surface. A further by-product of our solutions is that they also accommodate a variable pressure on the free surface which appears to be of relevance in modeling the event of storms in the ocean [50]. In the same strain, it seems that the adjustment of surface pressure conditions represents an important avenue leading to the appearance of critical layers [39].

Another remarkable feature of the solution is that the flow it prescribes accommodates a density stratification that varies (in the most general way) with depth. A noteworthy aspect is that the density is instrumental in conferring the velocity field a non-trivial vertical structure.

Density stratification has an essential role in geophysical fluid dynamics, particularly in the equatorial region where large scale ocean flows display marked density variations leading to a layering of the flow: fluid layers of different densities organize themselves so that the higher densities are found below lower densities, cf. [13, 16, 20]. Nevertheless, allowing for variable density significantly complicates the mathematical analysis of an already convoluted problem. Indeed, even in the simpler case of two-dimensional gravity water waves (without Coriolis effects) stratified fluids remained to a large extent unapproachable to a thorough mathematical analysis until recently: we refer the reader to [13, 16, 21, 28, 30–32, 43, 44, 47, 53, 55] for a selection of recent advances in the field. On a similar note we would like to remark the very recent results by Escher et al. [23] on stratified water waves exhibiting a very general density distribution allowing for singular gradients.

After a brief presentation of the water wave problem in Sect. 2 we indicate at the beginning of Sect. 3 the ansatz for the velocity field and we subsequently derive the formula for the pressure function satisfying the Euler’s equations. Afterwards, from the dynamic condition on the surface we determine an implicit equation for the surface defining function. Functional analytic methods are applied to the latter implicit equation to prove the existence of the surface defining function. Section 4 is concerned with the search for obtaining other (almost) solutions to the water wave problem, the latter task being achieved by means of a perturbation of the basic flow solutions (3.1). Although these perturbed solutions are not explicit they present bounded amplitude. To reach our goal we will avail of the short-wavelength perturbation method for three-dimensional flows, devised by Bayly [1], Friedlander and Vishik [24] and Lifschitz and Hameiri [41] and which examines whether the amplitude of the perturbations to the basic flow remains bounded as a function of time. The short-wavelength perturbation method emerged as an essential tool for boosting up the relevance of some recently derived exact solutions in geophysical fluid dynamics [11, 30–32, 34–36]. To be more specific, we start our attempt by searching for perturbations (of the velocity field and of the pressure) in the form of the Wentzel–Kramers–Brillouin (WKB) ansatz, cf. formula (4.1). As it turns out, the components of the amplitude vector of the perturbation to the basic flow satisfy a system of ordinary differential equations. We then prove that the latter system is equivalent to the Hill’s equation. A quite involved analysis is then used to show the boundedness of solutions to the Hill’s equation.

## The three-dimensional Euler equations with free surface boundary conditions

We formulate here the governing equations for free surface water flows. Working in a Cartesian coordinate system of coordinates *x*, *y*, *z*, we assume the water flow to be bounded below by the bed $z=-d$ and above by the free surface $z=\eta (x,y,t)$, where $\eta$ is a function that is determined as part of the solution and *t* denotes the time variable.

The guiding principles for water flow propagation refer to mass conservation and Newton’s second law of motion. The equation of mass conservation relates the rate of change of density to the field of motion. More precisely, denoting with $\rho =\rho (x,y,z,t)$ the density function the equation of mass conservation has the form

2.1 $$\begin{aligned} \rho _t+(\rho u)_x+(\rho v)_y+(\rho w)_z=0, \end{aligned}$$

where (*u*, *v*, *w*) represents the velocity field. Under the assumption that the water is inviscid, the equations of momentum conservation are

2.2 $$\begin{aligned} \begin{aligned} u_{t}+u u_{x}+v u_{y}+w u_{z}&= -\frac{1}{\rho }P_x,\\ v_{t}+u v_{x}+v v_{y}+w v_{z}&= -\frac{1}{\rho }P_y,\\ w_{t}+u w_{x}+v w_{y}+ w w_{z}&= -\frac{1}{\rho }P_z-g, \end{aligned} \end{aligned}$$

where *P* is the pressure function and *g* denotes the gravitational constant.

While both (2.1), (2.2) are required to hold within the bulk of the fluid, the specification of the water wave problem is completed by the boundary conditions pertaining to the free surface $z=\eta (x,y,t)$ and to the bed $z=-d$. These are the kinematic boundary conditions

2.3 $$\begin{aligned} w=\eta _t +u\eta _x+v\eta _y \quad \mathrm{on}\quad z=\eta (x,y,t) \end{aligned}$$

and

2.4 $$\begin{aligned} w=0\quad \mathrm{on}\quad z=-d, \end{aligned}$$

together with the dynamic boundary condition

2.5 $$\begin{aligned} P=p(x,y,t)\quad \mathrm{on}\quad z=\eta (x,y,t), \end{aligned}$$

for some given function $(x,y)\rightarrow p(x,y,t)$.

In the next section we introduce a family of solutions to the problem (2.2)–(2.5).

## A family of solutions exhibiting vertical structure

We first lay out the ansatz giving the explicit formulas of the velocity field and then, availing of the Euler’s equations (2.2), we derive the formula for the pressure function. The dynamic surface condition (2.5) will then be used to derive an implicit equation for the surface defining function. The latter equation is analysed by way of the implicit function theorem. More precisely, we will show that there is a unique function describing the shape of the surface, as soon as one applies on the surface a continuous pressure which is a small deviation from the pressure required to maintain a flat surface. In certain scenarios (defined by the choice of the density function) the free surface can also be determined explicitly. To begin with, let us set

3.1 $$\begin{aligned} u(x,y,z)=-\frac{yf(x^2+y^2)}{\sqrt{\rho (z)}},\quad v(x,y,z)=\frac{xf(x^2+y^2)}{\sqrt{\rho (z)}}\,\, \mathrm{and}\,\,w(x,y,z)=0, \end{aligned}$$

where $f:\mathbb {R}\rightarrow \mathbb {R}$ is such that the functions $(x,y)\rightarrow -yf(x^2+y^2)$ and $(x,y)\rightarrow x f(x^2+y^2)$ are differentiable and the density function $z\rightarrow \rho (z)$ (assumed here to depend only on the depth variable) is positive and differentiable. We would like to note that the velocity field given in (3.1) has a purely radial character with streamlines given by circles $x^2+y^2=k$ for some constant $k>0$.

A computation shows that

3.2 $$\begin{aligned} \begin{aligned} uu_x+vu_y=-\frac{xf^2(x^2+y^2)}{\rho (z)}\\ uv_x+vv_y=-\frac{yf^2(x^2+y^2)}{\rho (z)}. \end{aligned} \end{aligned}$$

This means that, in order to find the pressure *P* we have to solve the system

3.3 $$\begin{aligned} \begin{aligned} P_x(x,y,z,t)&=xf^2(x^2+y^2)\\ P_y(x,y,z,t)&=yf^2(x^2+y^2)\\ P_z(x,y,z,t)&=-g\rho . \end{aligned} \end{aligned}$$

Solving for *P* in the above system we obtain

3.4 $$\begin{aligned} P(x,y,z,t)=\frac{1}{2}\int _0^{x^2+y^2}f^2(s)ds-g\int _{-d}^z\rho (s)\,ds+c, \end{aligned}$$

for some constant *c*.

Clearly,

$$(\rho u)_x=-2xy\sqrt{\rho (z)}f^{\prime }(x^2+y^2),\quad (\rho v)_y=2xy\sqrt{\rho (z)}f^{\prime }(x^2+y^2),$$

which shows that equation of mass conservation is verified by the velocity field (3.1).

We set out to determine the free surface $\eta$ and to check the boundary conditions (2.3)–(2.5). Using the dynamic surface condition (2.5) the free surface $\eta$ is determined implicitly by the equation

3.5 $$\begin{aligned} \frac{1}{2}\int _0^{x^2+y^2}f^2(s)ds -g\int _{-d}^{\eta (x,y)}\rho (s)\,ds+c-p(x,y)=0. \end{aligned}$$

Let us recast the previous equation as the functional equation

3.6 $$\begin{aligned} \mathcal {F}(\eta ,p)=0, \end{aligned}$$

where

3.7 $$\begin{aligned} \mathcal {F}(\eta ,p):=\frac{1}{2}\int _0^{x^2+y^2}f^2(s)ds -g\int _{-d}^{\eta (x,y)}\rho (s)\,ds+c-p(x,y). \end{aligned}$$

Note that $\eta _0\equiv 0$ is a solution of (3.6) provided

$$\begin{aligned} p=p_0(x,y)=\frac{1}{2}\int _0^{x^2+y^2}f^2(s)ds -g\int _{-d}^{0}\rho (s)\,ds+c, \end{aligned}$$

that is $$\mathcal {F}(0,p_0)=0$$.

We will resort now to the implicit function theorem to prove the existence of $\eta$ satisfying (3.6). To this end we denote with $C_b(\mathbb {R}^2)$ the Banach space of continuous and bounded functions defined on $\mathbb {R}^2$. Note that

3.8 $$\begin{aligned} \mathcal {F}_{\eta }(0,p_0)(\eta )=-g\rho (0)\eta , \end{aligned}$$

which shows that $\mathcal {F}_{\eta }(0,p_0):C_b(\mathbb {R}^2)\rightarrow C_b(\mathbb {R}^2)$ is a homeomorphism. This implies that for all continuously prescribed surface pressure functions *p*(*x*, *y*), which represent a small deviation from the pressure $p_0$ required to maintain a flat free surface $\eta _0\equiv 0$, there is a unique continuous $\eta$ satisfying (3.5). The surface defining function $\eta$ is in fact differentiable as it can be derived from (3.5) by building the appropriate difference quotients and applying the mean value theorem. We obtain

3.9 $$\begin{aligned} \begin{aligned} g\rho (\eta (x,y))\eta _x=xf^2(x^2+y^2)-p_x\\ g\rho (\eta (x,y))\eta _y=yf^2(x^2+y^2)-p_y. \end{aligned} \end{aligned}$$

If the given pressure on the free surface is a function of type $p(x,y)=G(x^2+y^2)$ we see from (3.9) that $u\eta _x+v\eta _y=0$. The latter implies that the surface kinematic condition (2.3) is satisfied.

While, in general, is not possible to establish an explicit formula for the shape of the free surface $z=\eta (x,y)$, some special choices of density functions will render an easy determination of it. We present below some examples of such density functions, which are decreasing with depth, thus holding physical grounds.

### Remark 3.1

Let us assume that $\rho (z)=\frac{1}{(z+d+e^{-d})^k}$ where $k >0, k\ne 1$. We then have from (3.5)

$$\begin{aligned} \frac{1}{2}\int _0^{x^2+y^2}f^2(s)ds +c-p(x,y)=g\left( \frac{(\eta (x,y)+d+e^{-d})^{1-k} }{1-k}-\frac{e^{d(k-1)}}{1-k}\right) , \end{aligned}$$

that is

3.10 $$\begin{aligned} \eta (x,y)=\left[ e^{d(k-1)}+\frac{1-k}{g}\left( \frac{1}{2}\int _0^{x^2+y^2}f^2(s)\,ds -p(x,y)+c\right) \right] ^{\frac{1}{1-k}}-d-e^{-d}, \end{aligned}$$

formula which for $k>1$ makes sense only if *p*(*x*, *y*) is chosen such that

$$\begin{aligned} ge^{d(k-1)}+(1-k)\left( \frac{1}{2}\int _0^{x^2+y^2}f^2(s)\,ds -p(x,y)+c\right) >0, \end{aligned}$$

a condition that is easily verified as soon as the imposed pressure at the surface satisfies

3.11 $$\begin{aligned} p(x,y)>\left( \frac{1}{2}\int _0^{x^2+y^2}f^2(s)\,ds +c\right) -\frac{ge^{d(k-1)}}{k-1}. \end{aligned}$$

### Remark 3.2

Taking $k=1$ in the formula for $\rho$ from Remark 3.1 we get that $\eta$ is given as

3.12 $$\begin{aligned} \eta (x,y)=\exp \left( \frac{1}{g}\cdot \left( \frac{1}{2}\int _0^{x^2+y^2} f^2(s)\, ds +c-p(x,y)\right) -d\right) -d-e^{-d}. \end{aligned}$$

## Stability of the perturbations along the streamlines of the basic flow

To reinforce the relevance of the explicit solutions to the full nonlinear governing equations (2.1)–(2.5) we perform in this section a perturbation analysis of the basic flow solutions (3.1) along the streamlines of (3.1). The (linear) stability/instability of the basic flow refers to the boundedness/unboundedness of the amplitude of perturbations of the basic flow along the streamlines of the basic flow. Determining the stability of a flow is of paramount physical importance. Indeed, the stability in the sense defined above is equivalent to finding time-dependent (nearly) solutions to the water wave problem. Here, the term “nearly” refers to the obstruction by which the perturbations of the basic flow (3.1) fail to be exact solutions. More precisely, this obstruction is represented by the quadratic terms $(\mathbf {U}\cdot \nabla )\mathbf {U}$, often neglectable.

However, proving stability is a task that raises serious mathematical challenges. We choose to use the short-wavelength stability method developed by Bayly [1], Friedlander and Vishik [24] and Lifschitz and Hameiri [41]. As it turns out the components of the amplitude vector of the perturbation to the basic flow (*u*, *v*, *w*) from (3.1) satisfy a system of ordinary differential equations. The latter system is proved to be equivalent to the Hill’s equation. We then study the boundedness of solutions to the Hill’s equation by means of a criterion of Zukovskii, cf. [56].

### On the general setting

We consider perturbations $\mathbf {U}=(U,V,W)$ and $\mathbf {P}$ of the velocity field (*u*, *v*, *w*) given in (3.1) and of the pressure *P* given in (3.4), respectively. These perturbations are analyzed along the streamlines of the basic flow (*u*, *v*, *w*). To be more precise, we use for $\mathbf {U}$ and $\mathbf {P}$ the WKB ansatz

4.1 $$\begin{aligned} \begin{aligned} \mathbf {U}(t,x,y,z)=\mathbf {A}(t,x,y,z)e^{\frac{i}{\varepsilon }F(t,x,y,z)}+\mathcal {O}(\varepsilon )\\ \mathbf {P}(t,x,y,z)=\varepsilon B(t,x,y,z)e^{\frac{i}{\varepsilon }F(t,x,y,z)}+\mathcal {O}(\varepsilon ^2), \end{aligned} \end{aligned}$$

where ${\mathbf{A}}=(A_1,A_2,A_3)$ denotes the amplitude of the perturbation, *F* is a scalar function (called the phase of the perturbation) and $\varepsilon >0$ denotes a small parameter. We set as initial condition

4.2 $$\begin{aligned} \mathbf {U}_0:=\mathbf {U}(0,x,y,z)=\mathbf {A}(0,x,y,z)e^{\frac{i}{\varepsilon }F(0,x,y,z)}=:\mathbf {A}_0(x,y,z)e^{\frac{i}{\varepsilon }F_0(x,y,z)}. \end{aligned}$$

We say that the basic flow $\mathbf {u}=(u,v,w)$ is stable if the amplitude $\mathbf {A}$ remains uniformly bounded in time.

From the requirement that the perturbed quantities $\mathbf {U}+\mathbf {u}$ and $\mathbf {P}+P$ satisfy the equations of conservation of mass and of momentum (2.1) and (2.2), respectively, we obtain (by way of neglecting the quadratic term $(\mathbf {U}\cdot \nabla )\mathbf {U}$) the system

4.3 $$\begin{aligned} \begin{aligned} \mathbf {U}_t+(\mathbf {u}\cdot \nabla ) \mathbf {U} +(\mathbf {U}\cdot \nabla )\mathbf {u}=&-\frac{1}{\rho }\nabla \mathbf {P}\\ \text {div}(\rho \mathbf {U})=&0. \end{aligned} \end{aligned}$$

Using now the WKB ansatz (4.1) and identifying the coefficients of $\varepsilon ^{-1},\varepsilon ^0,\varepsilon ^1$ from (4.3) we obtain

4.4 $$\begin{aligned} \begin{aligned} A_{1,t}+uA_{1,x}+vA_{1,y}+wA_{1,z}+A_1u_x+A_2u_y+A_3u_z=-i\frac{B}{\rho }F_x,\\ A_{2,t}+uA_{2,x}+vA_{2,y}+wA_{2,z}+A_1v_x+A_2v_y+A_3v_z=-i\frac{B}{\rho }F_y,\\ A_{3,t}+uA_{3,x}+vA_{3,y}+wA_{3,z}+A_1w_x+A_2w_y+A_3w_z=-i\frac{B}{\rho }F_z.\\ \end{aligned} \end{aligned}$$

Moreover, *B* satisfies

4.5 $$\begin{aligned} B_x(t,x,y,z)=B_y(t,x,y,z)=B_z(t,x,y,z)=0, \end{aligned}$$

while the phase *F* is solution to the equation

4.6 $$\begin{aligned} F_t+uF_x+vF_y+wF_z=0, \end{aligned}$$

which, since $w=0$, reduces to

4.7 $$\begin{aligned} F_t+uF_x+vF_y=0. \end{aligned}$$

We proceed to solve (4.7) by the method of characteristics and so obtain the existence of a function $(X,Y,Z)\rightarrow \Theta (X,Y,Z)$ such that

4.8 $$\begin{aligned} \begin{aligned}&F(t,x,y,z)=\\&\Theta \big (x\cos (C(t-\overline{c}))+y\sin (C(t-\overline{c})),-x\sin (C(t-\overline{c}))+y\cos (C(t-\overline{c})),z\big ), \end{aligned} \end{aligned}$$

where $C=\frac{f(\tilde{c})}{\sqrt{\rho (c_3)}}$ and $\overline{c}$, $\tilde{c}$ and $c_3$ are some constants.

### Stability of the flows (3.1) for positive *f* and $f^{\prime }$

The purpose of the first part of this section is to show that there are choices for the phase function *F* for which the right hand side in system (4.4) vanishes. To begin with let us denote now with (*x*(*t*), *y*(*t*), *z*(*t*)) the streamlines of the basic flow (*u*, *v*, *w*) given in (3.1). This means that the equalities

4.9 $$\begin{aligned} \begin{aligned}&\frac{dx}{dt}=u(x(t),y(t),z(t))=-\frac{y(t) f(x^2(t)+y^2(t))}{\sqrt{\rho (z(t))}},\\&\frac{dy}{dt}=v(x(t),y(t),z(t))=\frac{x(t) f(x^2(t)+y^2(t))}{\sqrt{\rho (z(t))}},\\&\frac{dz}{dt}=w(x(t),y(t),z(t))=0, \end{aligned} \end{aligned}$$

hold for all *t*. Proceeding to solve (4.9) we infer first that there is a constant *c* such that

$$x^2(t)+y^2(t)=c\quad \mathrm{for}\,\,\mathrm{all}\quad t.$$

Consequently, we obtain the existence of constants $z_0, a_1$ and $a_2$ such that the equalities

4.10 $$\begin{aligned} \begin{aligned}&z(t)=z_0\\&x(t)=a_1\cos \left( \frac{f(c)}{\sqrt{\rho (z_0)}}t\right) +a_2\sin \left( \frac{f(c)}{\sqrt{\rho (z_0)}}t\right) ,\\&y(t)=a_1\sin \left( \frac{f(c)}{\sqrt{\rho (z_0)}}t\right) -a_2\cos \left( \frac{f(c)}{\sqrt{\rho (z_0)}}t\right) ,\\ \end{aligned} \end{aligned}$$

hold for all *t*. It follows that

4.11 $$\begin{aligned} \begin{aligned}&F_x(t,x(t),y(t),z(t))\\&\quad =\Theta _X\big (\mathbb {X}(t), \mathbb {Y}(t), \mathbb {Z}(t)\big )\cos (C(t-c)) -\Theta _Y\big (\mathbb {X}(t), \mathbb {Y}(t), \mathbb {Z}(t)\big )\sin (C(t-c)), \end{aligned} \end{aligned}$$

4.12 $$\begin{aligned} \begin{aligned}&F_y(t,x(t),y(t),z(t))\\&\quad =\Theta _X\big (\mathbb {X}(t), \mathbb {Y}(t), \mathbb {Z}(t)\big )\sin (C(t-c))+\Theta _Y\big (\mathbb {X}(t), \mathbb {Y}(t), \mathbb {Z}(t)\big )\cos (C(t-c)), \end{aligned} \end{aligned}$$

and

4.13 $$\begin{aligned} \begin{aligned}&F_z(t,x(t),y(t),z(t))=\Theta _Z\big (\mathbb {X}(t), \mathbb {Y}(t), \mathbb {Z}(t)\big ), \end{aligned} \end{aligned}$$

where

4.14 $$\begin{aligned} \begin{aligned} \mathbb {X}(t):&=x(t)\cos (C(t-c))+y(t)\sin (C(t-c))\equiv a_1\cos (c)+a_2\sin (c), \\ \mathbb {Y}(t):&=-x(t)\sin (C(t-c))+y(t)\cos (C(t-c)) \equiv a_1\sin (c)-a_2\cos (c),\\ \mathbb {Z}(t):&=z(t)\equiv z_0. \end{aligned} \end{aligned}$$

After picking a certain streamline (*x*(*t*), *y*(*t*), *z*(*t*)) of the basic flow (*u*, *v*, *w*) (by assigning initial values $z_0, a_1,a_2$ for *x*(*t*), *y*(*t*), *z*(*t*) in (4.10)) we choose $\Theta$ in the expression of the phase *F* of the perturbation such that

4.15 $$\begin{aligned} \begin{aligned}&\Theta _X \left( a_1\cos (c)+a_2\sin (c), a_1\sin (c)-a_2\cos (c), z_0\right) =0,\\&\Theta _Y \left( a_1\cos (c)+a_2\sin (c), a_1\sin (c)-a_2\cos (c), z_0\right) =0,\\&\Theta _Z \left( a_1\cos (c)+a_2\sin (c), a_1\sin (c)-a_2\cos (c), z_0\right) =0. \end{aligned} \end{aligned}$$

Inserting the streamlines (4.10) of the basic flow

$$\begin{aligned} u(x,y,z)=-\frac{yf(x^2+y^2)}{\sqrt{\rho (z)}},\quad v(x,y,z)=\frac{xf(x^2+y^2)}{\sqrt{\rho (z)}}, \quad w(x,y,z)\equiv 0 \end{aligned}$$

into the system (4.4) we obtain (taking also into account the choice (4.15)) that the amplitude triple $(A_1,A_2,A_3)$ satisfies the homogeneous system

4.16 $$\begin{aligned} \begin{pmatrix} \frac{d}{dt}\big (A_1(t,x(t),y(t),z(t)\big )\\ \\ \frac{d}{dt}\big (A_2(t,x(t),y(t),z(t)\big )\\ \\ \frac{d}{dt}\big (A_3(t,x(t),y(t),z(t)\big )\\ \end{pmatrix} =\mathcal {M}(t) \begin{pmatrix} A_1(t,x(t),y(t),z(t)\\ \\ A_2(t,x(t),y(t),z(t)\\ \\ A_3(t,x(t),y(t),z(t) \end{pmatrix} \end{aligned}$$

where

4.17 $$\begin{aligned} \mathcal {M}(t)=-\begin{pmatrix} \frac{-2x(t)y(t)f^{\prime }(c)}{\sqrt{\rho (z_0)}} &{}\quad -\frac{f(c)+2y^2(t)f^{\prime }(c)}{\sqrt{\rho (z_0)}} &{}\quad \frac{y(t)\rho ^{\prime }(z_0)f(c)}{2\rho (z_0)^{\frac{3}{2}}}\\ &{} &{}\\ \frac{f(c)+2x^2(t)f^{\prime }(c)}{\sqrt{\rho (z_0)}} &{}\quad \frac{2x(t)y(t)f^{\prime }(c)}{\sqrt{\rho (z_0)}} &{}\quad \frac{-x(t)\rho ^{\prime }(z_0)f(c)}{2\rho (z_0)^{\frac{3}{2}}}\\ &{} &{}\\ 0 &{}\quad 0 &{}\quad 0 \end{pmatrix}. \end{aligned}$$

More explicitly, denoting $A_i(t,x(t),y(t),z(t))=\tilde{A}_i(t)$ we obtain from the previous system that

4.18 $$\begin{aligned} \begin{aligned}&\tilde{A}_3(t) = \tilde{A}_3(0)=:\beta \,\,\mathrm{for}\,\,\mathrm{all}\,\,t\\&\begin{pmatrix}\tilde{A}_1^{\prime }(t) \\ \tilde{A}_2^{\prime }(t)\end{pmatrix}=M(t)\begin{pmatrix} \tilde{A}_1(t) \\ \tilde{A}_2(t)\end{pmatrix}+\begin{pmatrix}-\alpha \beta y(t)\\ \alpha \beta x(t)\end{pmatrix}, \end{aligned} \end{aligned}$$

where

4.19 $$\begin{aligned} M(t)= \begin{pmatrix} \frac{2x(t)y(t)f^{\prime }(c)}{\sqrt{\rho (z_0)}} &{}\quad \frac{f(c)+2y^2(t)f^{\prime }(c)}{\sqrt{\rho (z_0)}}\\ -\frac{f(c)+2x^2(t)f^{\prime }(c)}{\sqrt{\rho (z_0)}} &{}\quad \frac{-2x(t)y(t)f^{\prime }(c)}{\sqrt{\rho (z_0)}} \end{pmatrix} \end{aligned}$$

and $\alpha :=\frac{\rho ^{\prime }(z_0)f(c)}{2\rho (z_0)^{\frac{3}{2}} }$. We will prove in the sequel that $\tilde{A}_1,\tilde{A}_2$ are bounded in *t*. We will be concerned first with the homogeneous system

4.20 $$\begin{aligned} \begin{pmatrix}\tilde{A}_1^{\prime }(t) \\ \tilde{A}_2^{\prime }(t)\end{pmatrix}=M(t)\begin{pmatrix} \tilde{A}_1(t) \\ \tilde{A}_2(t)\end{pmatrix}, \end{aligned}$$

which can be equivalently written as

4.21 $$\begin{aligned} \begin{aligned} \frac{d}{dt}\left( \tilde{A}_1(t) e^{-\int _0^t \frac{2x(s)y(s)f^{\prime }(c)}{ \sqrt{\rho (z_0)}} \,ds }\right) = \frac{(f(c)+2y^2(t) f^{\prime }(c))}{ \sqrt{\rho (z_0)} } \tilde{A}_2(t)e^{-\int _0^t \frac{2x(s)y(s)f^{\prime }(c)}{ \sqrt{\rho (z_0)}} \,ds }\\ \frac{d}{dt}\left( \tilde{A}_2(t) e^{\int _0^t \frac{2x(s)y(s)f^{\prime }(c)}{ \sqrt{\rho (z_0)}} \,ds }\right) =-\frac{(f(c)+2x^2(t) f^{\prime }(c))}{ \sqrt{\rho (z_0)}}\tilde{A}_1(t)e^{\int _0^t \frac{2x(s)y(s)f^{\prime }(c)}{ \sqrt{\rho (z_0)}}\,ds }. \end{aligned} \end{aligned}$$

Denoting

4.22 $$\begin{aligned} \bar{A}_1:=\tilde{A}_1(t) e^{-\int _0^t \frac{2x(s)y(s)f^{\prime }(c)}{ \sqrt{\rho (z_0)}}\,ds},\quad \bar{A}_2:=\tilde{A}_2(t) e^{\int _0^t \frac{2x(s)y(s)f^{\prime }(c)}{ \sqrt{\rho (z_0)}}\,ds }, \end{aligned}$$

we see that the previous system can be written as

4.23 $$\begin{aligned} \begin{aligned} \frac{d\bar{A}_1}{dt}=\frac{(f(c)+2y^2(t)f^{\prime }(c))}{ \sqrt{\rho (z_0)}} e^{-\int _0^t \frac{4x(s)y(s)f^{\prime }(c)}{ \sqrt{\rho (z_0)}}\,ds }\bar{A}_2\\ \frac{d\bar{A}_2}{dt}=-\frac{(f(c)+2x^2(t) f^{\prime }(c))}{ \sqrt{\rho (z_0)}} e^{\int _0^t \frac{4x(s)y(s)f^{\prime }(c)}{ \sqrt{\rho (z_0)}}\,ds }\bar{A}_1. \end{aligned} \end{aligned}$$

Eliminating $\bar{A}_2$ from above we get that $\bar{A}_1$ satisfies the equation

4.24 $$\begin{aligned} \frac{d}{dt}\left( \frac{ \sqrt{\rho (z_0)} e^{\int _0^t \frac{4x(s)y(s)f^{\prime }(c)}{ \sqrt{\rho (z_0)} } \,ds }}{f(c)+2y^2(t) f^{\prime }(c)}\cdot \frac{d\bar{A}_1}{dt}\right) =-\frac{(f(c)+2x^2(t) f^{\prime }(c))}{ \sqrt{\rho (z_0)} } e^{\int _0^t \frac{4x(s)y(s)f^{\prime }(c)}{ \sqrt{\rho (z_0)} } \,ds }\bar{A}_1, \end{aligned}$$

while, $$\bar{A}_2$$ verifies

4.25 $$\begin{aligned} \frac{d}{dt}\left( \frac{ \sqrt{\rho (z_0)} e^{-\int _0^t \frac{4x(s)y(s)f^{\prime }(c)}{ \sqrt{\rho (z_0)} } \,ds }}{f(c)+2x^2(t) f^{\prime }(c)}\cdot \frac{d\bar{A}_2}{dt}\right) =-\frac{(f(c)+2y^2(t) f^{\prime }(c))}{ \sqrt{\rho (z_0)} } e^{-\int _0^t \frac{4x(s)y(s)f^{\prime }(c)}{ \sqrt{\rho (z_0)} } \,ds }\bar{A}_2. \end{aligned}$$

Upon setting

4.26 $$\begin{aligned} p_1(t):= \frac{ \sqrt{\rho (z_0)} e^{\int _0^t \frac{4x(s)y(s)f^{\prime }(c)}{ \sqrt{\rho (z_0)} } \,ds }}{f(c)+2y^2(t) f^{\prime }(c)},\,\,\,\, q_1(t):=\frac{(f(c)+2x^2(t) f^{\prime }(c))}{ \sqrt{\rho (z_0)} } e^{\int _0^t \frac{4x(s)y(s)f^{\prime }(c)}{ \sqrt{\rho (z_0)} } \,ds }, \end{aligned}$$

we obtain

4.27 $$\begin{aligned} \frac{d^2\bar{A}_1}{dt^2}+\frac{p_1^{\prime }(t)}{p_1(t)}\frac{d\bar{A}_1}{dt}+\frac{q_1(t)}{p_1(t)}\bar{A}_1=0. \end{aligned}$$

With the substitution

4.28 $$\begin{aligned} \hat{A}_1:=\sqrt{p_1(t)}\bar{A}_1 \end{aligned}$$

we can convert (4.27) into the Hill’s equation

4.29 $$\begin{aligned} \frac{d^2 \hat{A}_1}{dt^2}+Q_1(t)\hat{A}_1=0 \end{aligned}$$

for

$$\begin{aligned} Q_1(t)=-\frac{1}{2}\cdot \frac{d}{dt}\left( \frac{p_1^{\prime }(t)}{p_1(t)}\right) -\frac{1}{4}\left( \frac{p_1^{\prime }(t)}{p_1(t)}\right) ^2+\frac{q_1(t)}{p_1(t)}. \end{aligned}$$

Moreover, setting

4.30 $$\begin{aligned} p_2(t):= \frac{ \sqrt{\rho (z_0)} e^{-\int _0^t \frac{4x(s)y(s)f^{\prime }(c)}{ \sqrt{\rho (z_0)} } \,ds }}{f(c)+2x^2(t) f^{\prime }(c)},\,\,\,\, q_2(t):=\frac{(f(c)+2y^2(t) f^{\prime }(c))}{ \sqrt{\rho (z_0)} } e^{-\int _0^t \frac{4x(s)y(s)f^{\prime }(c)}{ \sqrt{\rho (z_0)} } \,ds }, \end{aligned}$$

we obtain

4.31 $$\begin{aligned} \frac{d^2\bar{A}_2}{dt^2}+\frac{p_2^{\prime }(t)}{p_2(t)}\frac{d\bar{A}_2}{dt}+\frac{q_2(t)}{p_2(t)}\bar{A}_2=0. \end{aligned}$$

Substituting

4.32 $$\begin{aligned} \hat{A}_2:=\sqrt{p_2(t)}\bar{A}_2 \end{aligned}$$

we can convert (4.31) into the Hill’s equation

4.33 $$\begin{aligned} \frac{d^2 \hat{A}_2}{dt^2}+Q_2(t)\hat{A}_2=0 \end{aligned}$$

for

$$\begin{aligned} Q_2(t)=-\frac{1}{2}\cdot \frac{d}{dt}\left( \frac{p_2^{\prime }(t)}{p_2(t)}\right) -\frac{1}{4}\left( \frac{p_2^{\prime }(t)}{p_2(t)}\right) ^2+\frac{q_2(t)}{p_2(t)}. \end{aligned}$$

Answering the question of boundedness of solutions to the Hill’s equation is a result of Zukovskii which we formulate below and refer the reader to [3, 56] for details.

#### Theorem 4.1

[56] Assume that $t\rightarrow Q(t)$ is a continuous periodic function (of period *L*). Then all solutions *y* of the Hill’s equation

$$\begin{aligned} y''+Q(t)y=0 \end{aligned}$$

are bounded if there exists some $n\in \mathbb {N}$ with the property that the inequality

4.34 $$\begin{aligned} n^2\left( \frac{\pi }{L}\right) ^2\le Q(t)\le (n+1)^2 \left( \frac{\pi }{L}\right) ^2 \end{aligned}$$

holds for all *t*.

#### Lemma 4.2

Let $L:= \pi \frac{\sqrt{\rho (z_0)}}{f(c)}$ be the (principal) period of $Q_1$ and choose constants $a_1,a_2$ in formula (4.10) such that

$$(a_1^2+a_2^2)\cdot \frac{f'(c)}{f(c)}\le \frac{1}{5}.$$

Then the inequality

4.35 $$\begin{aligned} \left( \frac{\pi }{L}\right) ^2< Q_1(t)< 3\left( \frac{\pi }{L}\right) ^2 \end{aligned}$$

holds for all *t*, provided the functions *f* and $f'$ are positive.

#### Proof

Taking into account formula (4.26) we obtain (after tedious calculations and some algebraic manipulations) that

4.36 $$\begin{aligned} \begin{aligned} Q_1(t)&=\frac{f^2(c)}{\rho (z_0)}\left[ 1+2y^2(t)\frac{f'(c)}{f(c)}+4y^4(t)\left( \frac{f'(c)}{f(c)}\right) ^2\cdot \frac{1}{1+2y^2(t)\frac{f'(c)}{f(c)}}\right] \\&\quad +\frac{f^2(c)}{\rho (z_0)}\cdot \frac{2x^2(t)\frac{f'(c)}{f(c)}\left[ 1+8y^4(t)\left( \frac{f'(c)}{f(c)}\right) ^2\right] }{\left( 1+2y^2(t)\frac{f'(c)}{f(c)}\right) ^2}. \end{aligned} \end{aligned}$$

To check condition (4.34) from Theorem 4.1 we remark that $Q_1$ is periodic of period $\pi \frac{\sqrt{\rho (z_0)}}{f(c)}=:L$. Acting on the assumption that *f* and $f'$ are positive functions we clearly have that

4.37 $$\begin{aligned} Q_1(t)>\frac{f^2(c)}{\rho (z_0)}=\left( \frac{\pi }{L}\right) ^2. \end{aligned}$$

Moreover, we also see that for all *t* holds

4.38 $$\begin{aligned} Q_1(t)<\frac{f^2(c)}{\rho (z_0)}\left[ 1+2y^2(t)\frac{f'(c)}{f(c)}+4y^4(t)\left( \frac{f'(c)}{f(c)}\right) ^2 +4x^2(t)\frac{f'(c)}{f(c)} \right] , \end{aligned}$$

where, to obtain the last term in the bracket above, we have used that

$$\begin{aligned} 1+8y^4(t)\left( \frac{f'(c)}{f(c)}\right) ^2<2\left( 1+2y^2(t)\frac{f'(c)}{f(c)}\right) ^2. \end{aligned}$$

In the quest to establish the bound on the right hand side of (4.35) we will seek to find $a_1, a_2$ (from the formula (4.10) defining the trajectories of the basic flow) such that the inequalities

4.39 $$\begin{aligned} y^2(t)\frac{f'(c)}{f(c)}\le \frac{1}{5}\quad \mathrm{and}\quad x^2(t)\frac{f'(c)}{f(c)}\le \frac{1}{5} \end{aligned}$$

hold for all *t*. From (4.38) and (4.39) we conclude

4.40 $$\begin{aligned} Q_1(t)<\frac{f^2(c)}{\rho (z_0)}\left( 1+\frac{2}{5}+\frac{4}{25}+\frac{4}{5}\right) <3\cdot \frac{f^2(c)}{\rho (z_0)}. \end{aligned}$$

Inequalities (4.37) and (4.40) can be restated as

$$\begin{aligned} \left( \frac{\pi }{L}\right) ^2<Q_1(t)<3\left( \frac{\pi }{L}\right) ^2\quad \mathrm{for}\,\,\mathrm{all}\,\,t, \end{aligned}$$

that is, we have proved (4.35) which guarantees the boundedness of solutions to (4.29).

To ensure that (4.39) holds we impose the conditions

4.41 $$\begin{aligned} \sup _{t\in \mathbb {R}}x^2(t)\frac{f'(c)}{f(c)}\le \frac{1}{5},\quad \sup _{t\in \mathbb {R}}y^2(t)\frac{f'(c)}{f(c)}\le \frac{1}{5}, \end{aligned}$$

Taking into account that

4.42 $$\begin{aligned} \begin{aligned} 2x^2(t)=a_1^2+a_2^2 +(a_1^2-a_2^2)\cos \left( \frac{2f(c)}{\sqrt{\rho (z_0)}}t\right) +a_1 a_2 \sin \left( \frac{2f(c)}{\sqrt{\rho (z_0)}}t\right) ,\\ 2y^2(t)=a_1^2+a_2^2 +(a_2^2-a_1^2)\cos \left( \frac{2f(c)}{\sqrt{\rho (z_0)}}t\right) -a_1 a_2 \sin \left( \frac{2f(c)}{\sqrt{\rho (z_0)}}t\right) , \end{aligned} \end{aligned}$$

we obtain

4.43 $$\begin{aligned} \begin{aligned} \sup _{t\in \mathbb {R}}x^2(t)\frac{f'(c)}{f(c)}&=\sup _{t\in \mathbb {R}}y^2(t)\frac{f'(c)}{f(c)}\\&=\frac{a_1^2+a_2^2+\sqrt{(a_1^2-a_2^2)^2+a_1^2 a_2^2}}{2}\cdot \frac{f'(c)}{f(c)}=:M.\\ \end{aligned} \end{aligned}$$

Hence, inequalities (4.41) are equivalent with

4.44 $$\begin{aligned} M\le \frac{1}{5}. \end{aligned}$$

Under the condition

$$(a_1^2+a_2^2)\frac{f'(c)}{f(c)}\le \frac{2}{5},$$

we can square in inequality (4.44)and obtain that (4.44) is satisfied precisely when

4.45 $$\begin{aligned} \begin{aligned} (a_1^2+a_2^2)\cdot \frac{f'(c)}{f(c)}&\le \frac{2}{5}\\ 4(a_1^2+a_2^2)\cdot \frac{f'(c)}{f(c)}&\le \frac{4}{5}+15a_1^2 a_2^2\left( \frac{f'(c)}{f(c)}\right) ^2 \end{aligned} \end{aligned}$$

where, we recall, $c=a_1^2+a_2^2$. Obviously, any $(a_1,a_2)$ with $(a_1^2+a_2^2)\cdot \frac{f'(c)}{f(c)}\le \frac{1}{5}$ also satisfies the second inequality in the latter system. $\square$

Analogously, we have

#### Lemma 4.3

Let $a_1,a_2$ from formula (4.10) be such that

$$(a_1^2+a_2^2)\cdot \frac{f'(c)}{f(c)}\le \frac{1}{5}.$$

Then the inequality

4.46 $$\begin{aligned} \left( \frac{\pi }{L}\right) ^2< Q_2(t)< 3\left( \frac{\pi }{L}\right) ^2 \end{aligned}$$

holds for all *t*, provided the functions *f* and $f'$ are positive.

#### Proof

A computation similar to the one in Lemma 4.2 shows that

4.47 $$\begin{aligned} \begin{aligned} Q_2(t)&=\frac{f^2(c)}{\rho (z_0)}\left[ 1+2x^2(t)\frac{f'(c)}{f(c)}+4x^4(t)\left( \frac{f'(c)}{f(c)}\right) ^2\cdot \frac{1}{1+2x^2(t)\frac{f'(c)}{f(c)}}\right] \\&\quad +\frac{f^2(c)}{\rho (z_0)}\cdot \frac{2y^2(t)\frac{f'(c)}{f(c)}\left[ 1+8x^4(t)\left( \frac{f'(c)}{f(c)}\right) ^2\right] }{\left( 1+2x^2(t)\frac{f'(c)}{f(c)}\right) ^2}. \end{aligned} \end{aligned}$$

Undertaking now an analysis similar to the one in Lemma 4.3 by interchanging the role *x*(*t*) and *y*(*t*) we obtain the claim (4.46). $\square$

#### Remark 4.4

Lemma (4.2) and Lemma 4.3 show via Theorem 4.1 that all the solutions of the two Hill equations (4.29) and (4.33) are bounded. Availing now of the boundedness of the functions $p_1,p_2, t\rightarrow \int _0^t \frac{2x(s)y(s)f^{\prime }(c)}{ \sqrt{\rho (z_0)}}\,ds$ and of the formulas (4.22), (4.28),(4.32) we infer now that all solutions $$\begin{pmatrix}\tilde{A}_1\\ \tilde{A}_2\end{pmatrix}$$ of the homogeneous system (4.20) are bounded.

#### Lemma 4.5

Let $t\rightarrow (x(t),y(t), z(t))$ be trajectories of the basic flow (3.1) given by (4.10) where the coefficients $a_2, a_2$ are chosen so that $(a_1^2+a_2^2)\cdot \frac{f'(c)}{f(c)}\le \frac{1}{5}$, where, $c=a_1^2+a_2^2$. Then all solutions of the system (4.18), and thus, all solutions of the system (4.16) are bounded.

#### Proof

We will seek to find a particular solution $$\begin{pmatrix}\tilde{A}_1\\ \tilde{A}_2\end{pmatrix}_p$$of the system (4.18) that is bounded. Then, any solution of (4.18) is bounded since it is obtained as the sum between a solution of the homogeneous system (4.20) and the particular solution $$\begin{pmatrix}\tilde{A}_1\\ \tilde{A}_2\end{pmatrix}_p$$. To this end, we make the ansatz

4.48 $$\begin{aligned} \begin{pmatrix}\tilde{A}_1\\ \tilde{A}_2\end{pmatrix}_p=\begin{pmatrix} \mathcal {A}\cos \left( \frac{f(c)t}{\sqrt{\rho (z_0)}}\right) +\mathcal {B}\sin \left( \frac{f(c)t}{\sqrt{\rho (z_0)}}\right) \\ -\mathcal {B}\cos \left( \frac{f(c)t}{\sqrt{\rho (z_0)}}\right) +\mathcal {A}\sin \left( \frac{f(c)t}{\sqrt{\rho (z_0)}}\right) \end{pmatrix}, \end{aligned}$$

where $\mathcal {A},\mathcal {B}$ are to be determined so that $$\begin{pmatrix}\tilde{A}_1\\ \tilde{A}_2\end{pmatrix}_p$$ satisfies (4.18). Routine calculations show that the first equation of the system (4.18) can be written as

4.49 $$\begin{aligned} \begin{aligned}&\left[ \frac{f'(c)}{\sqrt{\rho (z_0)}}\left( (a_1^2-a_2^2+c)\mathcal {A}+2a_1a_2\mathcal {B}\right) +\frac{2f(c)}{ \sqrt{\rho (z_0)} } \mathcal {A}\right] \sin \left( \frac{f(c)t}{\sqrt{\rho (z_0)}}\right) \\&\qquad +\left[ \frac{f'(c)}{\sqrt{\rho (z_0)}}\left( (a_1^2-a_2^2-c)\mathcal {B}-2a_1 a_2\mathcal {A}\right) -\frac{2f(c)}{ \sqrt{\rho (z_0)} } \mathcal {B}\right] \cos \left( \frac{f(c)t}{\sqrt{\rho (z_0)}}\right) \\&\quad =\alpha \beta y(t)=\alpha \beta \left[ a_1\sin \left( \frac{f(c)t}{\sqrt{\rho (z_0)}}\right) -a_2\cos \left( \frac{f(c)t}{\sqrt{\rho (z_0)}}\right) \right] , \end{aligned} \end{aligned}$$

which, after identification of the coefficients of $\sin \left( \frac{f(c)t}{\sqrt{\rho (z_0)}}\right)$ and $\cos \left( \frac{f(c)t}{\sqrt{\rho (z_0)}}\right)$, respectively, is equivalent with the system in the unknowns $\mathcal {A},\mathcal {B}$

4.50 $$\begin{aligned} \begin{aligned}&\frac{2a_1^2 f'(c)+2f(c)}{\sqrt{\rho (z_0)}}\mathcal {A}+\frac{2a_1 a_2 f'(c)}{\sqrt{\rho (z_0)}}\mathcal {B}=\alpha \beta a_1\\&\frac{2a_1 a_2 f'(c)}{\sqrt{\rho (z_0)}}\mathcal {A}+\frac{2a_2^2 f'(c)+2f(c)}{\sqrt{\rho (z_0)}}\mathcal {B}=\alpha \beta a_2. \end{aligned} \end{aligned}$$

Analogously, we find that the second equation of (4.18) becomes

4.51 $$\begin{aligned} \begin{aligned}&\left[ \frac{f'(c)}{\sqrt{\rho (z_0)}}\left( (a_1^2-a_2^2-c)\mathcal {B}-2 a_1 a_2\mathcal {A}\right) -\frac{2f(c)}{\sqrt{\rho (z_0)}}\mathcal {B} \right] \sin \left( \frac{f(c)t}{\sqrt{\rho (z_0)}}\right) \\&\qquad +\left[ \frac{f'(c)}{\sqrt{\rho (z_0)}}\left( (a_2^2-a_1^2 -c)\mathcal {A}-2a_1 a_2 \mathcal {B}\right) -\frac{2f(c)}{\sqrt{\rho (z_0)}}\mathcal {A} \right] \cos \left( \frac{f(c)t}{\sqrt{\rho (z_0)}}\right) \\&\quad =-\alpha \beta x(t)=-\alpha \beta \left[ a_1 \cos \left( \frac{f(c)t}{\sqrt{\rho (z_0)}}\right) +a_2 \sin \left( \frac{f(c)t}{\sqrt{\rho (z_0)}}\right) \right] . \end{aligned} \end{aligned}$$

Identifying the coefficients of $\sin \left( \frac{f(c)t}{\sqrt{\rho (z_0)}}\right)$ and $\cos \left( \frac{f(c)t}{\sqrt{\rho (z_0)}}\right)$, respectively, we obtain that $\mathcal {A}$ and $\mathcal {B}$ satisfy the system

4.52 $$\begin{aligned} \begin{aligned}&\frac{ 2a_1 a_2 f'(c)}{\sqrt{\rho (z_0)}}\mathcal {A}+\frac{2a_2^2 f'(c) +2f(c)}{\sqrt{\rho (z_0)}}\mathcal {B}=\alpha \beta a_2\\&\frac{2 a_1^2 f'(c)+2f(c)}{\sqrt{\rho (z_0)}}\mathcal {A}+\frac{2 a_1 a_2 f'(c)}{\sqrt{\rho (z_0)}}\mathcal {B} =\alpha \beta a_1, \end{aligned} \end{aligned}$$

which coincides with the system (4.50). From the previous considerations we can draw the conclusion that the ansatz $$\begin{pmatrix}\tilde{A}_1\\ \tilde{A}_2\end{pmatrix}_p$$ from (4.48) represents a particular solution of (4.18) if and only $(\mathcal {A}, \mathcal {B})$ is a solution of (4.52). We remark that the determinant of the matrix of the system in (4.52) equals $\frac{-4f^2(c)-4cf(c)f'(c)}{\rho (z_0)}<0$ since *f*(*c*) and $f'(c)$ were assumed to be positive. Hence, there are unique $\mathcal {A},\mathcal {B}$ that verify (4.52), assertion equivalent to the existence of a unique solution to (4.18) of the type (4.48). Nevertheless, this suffices for our purposes. $\square$

We state now the main result on the stability of perturbations along the streamlines of the basic flow (3.1).

#### Theorem 4.6

Let us consider the flow

4.53 $$\begin{aligned} u(x,y,z)=-\frac{yf(x^2+y^2)}{\sqrt{\rho (z)}},\quad v(x,y,z)=\frac{xf(x^2+y^2)}{\sqrt{\rho (z)}}\,\, \mathrm{and}\,\,w(x,y,z)=0, \end{aligned}$$

with positive *f* and $f'$ and let

4.54a $$\begin{aligned} \begin{aligned}&x(t)=a_1\cos \left( \frac{f(c)}{\sqrt{\rho (z_0)}}t\right) +a_2\sin \left( \frac{f(c)}{\sqrt{\rho (z_0)}}t\right) ,\\&y(t)=a_1\sin \left( \frac{f(c)}{\sqrt{\rho (z_0)}}t\right) -a_2\cos \left( \frac{f(c)}{\sqrt{\rho (z_0)}}t\right) ,\\&z(t)=z_0,\\ \end{aligned} \end{aligned}$$

be trajectories of (4.53), where $a_1,a_2$ satisfy

4.54b $$\begin{aligned} (a_1^2+a_2^2)\cdot \frac{f'(c)}{f(c)}\le \frac{1}{5}. \end{aligned}$$

Then, regardless of the density $\rho$, the flow (4.53) is (linearly) stable under the short-wavelength perturbations (4.1) along the streamlines () provided the phase *F* of the perturbation is constant or satisfies (4.15).

### On the stability of some particular flows

We address now the stability issue of the basic flow (3.1) for the situation when the function *f* from the definition of *u*, *v* is equal to 1. In this case it is possible to derive explicit formulas for the trajectories of the amplitudes of the trajectories of the basic flow. More precisely, we easily see that the amplitudes $\tilde{A}_1,\tilde{A}_2,\tilde{A}_3$ verify the system

4.55 $$\begin{aligned} \begin{aligned}&\tilde{A}_3(t)\equiv \tilde{A}_3(0)=:\beta ,\\&\tilde{A}_1^{\prime }(t)=\frac{1}{\sqrt{\rho (z_0)}}\tilde{A}_2(t)-\alpha \beta y(t),\\&\tilde{A}_2^{\prime }(t)=-\frac{1}{\sqrt{\rho (z_0)}}\tilde{A}_1(t)+\alpha \beta x(t), \end{aligned} \end{aligned}$$

where $\alpha :=\frac{\rho ^{\prime }(z_0)}{2\rho (z_0)^{\frac{3}{2}} }$. From (4.18) and appealing also to (4.9) we find that

4.56 $$\begin{aligned} \begin{aligned} \tilde{A}_1^{\prime \prime }(t)=-\frac{1}{\rho (z_0)}\tilde{A}_1(t)+\frac{\alpha \beta }{\sqrt{\rho (z_0)}}x(t)-\alpha \beta y^{\prime }(t)=-\frac{1}{\rho (z_0)}\tilde{A}_1(t),\\ \tilde{A}_2^{\prime \prime }(t)=-\frac{1}{\rho (z_0)}\tilde{A}_2(t)+\frac{\alpha \beta }{\sqrt{\rho (z_0)}}y(t)+\alpha \beta x^{\prime }(t)=-\frac{1}{\rho (z_0)}\tilde{A}_2(t). \end{aligned} \end{aligned}$$

Therefore, there are constants $\gamma _1,\gamma _2$ and $\theta _1,\theta _2$ such that

4.57 $$\begin{aligned} \begin{aligned} \tilde{A}_1(t)=\gamma _1\cos \left( \frac{t}{\sqrt{\rho (z_0)}}\right) +\gamma _2\sin \left( \frac{t}{\sqrt{\rho (z_0)}}\right) \\ \tilde{A}_2(t)=\theta _1\cos \left( \frac{t}{\sqrt{\rho (z_0)}}\right) +\theta _2\sin \left( \frac{t}{\sqrt{\rho (z_0)}}\right) , \end{aligned} \end{aligned}$$

from which we see that $\tilde{A}_1$ and $\tilde{A}_2$ are clearly bounded. We therefore have the following result.

#### Theorem 4.7

The flow

4.58 $$\begin{aligned} u(x,y,z)=\frac{-y}{\sqrt{\rho (z)}},\quad v(x,y,z)=\frac{x}{\sqrt{\rho (z)}}\,\, \mathrm{and}\,\,w(x,y,z)=0 \end{aligned}$$

is stable under the short-wavelength perturbations (4.1) along the streamlines of the basic flow

4.59 $$\begin{aligned} \begin{aligned}&x(t)=a_1\cos \left( \frac{t}{\sqrt{\rho (z_0)}}\right) +a_2\sin \left( \frac{t}{\sqrt{\rho (z_0)}}\right) ,\\&y(t)=a_1\sin \left( \frac{t}{\sqrt{\rho (z_0)}}\right) -a_2\cos \left( \frac{t}{\sqrt{\rho (z_0)}}\right) ,\\&z(t)=z_0,\\ \end{aligned} \end{aligned}$$

provided the phase *F* of the perturbation (4.1) is constant or satisfies (4.15).

## Data Availability

My manuscript has no associated data.
